# Survival improvement in primary plasma cell leukemia: a retrospective analysis of novel agent-based regimens and stem cell transplantation

**DOI:** 10.3389/fonc.2025.1727117

**Published:** 2026-01-09

**Authors:** Yong Xu, Jie He, Lanxin Chen, Jinwen Liu, Min Zhou, Bing Chen, Hua Bai

**Affiliations:** Department of Hematology, Nanjing Drum Tower Hospital, Affiliated Hospital of Medical School, Nanjing University, Nanjing, China

**Keywords:** autologous stem cell transplantation, primary plasma cell leukemia, prognostic factors, treatment response, venetoclax

## Abstract

**Background:**

This study aimed to characterize the clinical profile and treatment outcomes of primary plasma cell leukemia (pPCL) patients, defined by ≥5% circulating plasma cells (CPCs) on peripheral blood smear, who received novel agent-based induction therapy.

**Methods:**

A retrospective analysis of 46 pPCL patients treated at Nanjing Drum Tower Hospital from March 2014 to April 2025 was conducted. Their clinical and laboratory manifestations, prognostic factors, and efficacy of induction therapies were focused upon.

**Results:**

Advanced-stage disease predominated in our cohort. The most frequent cytogenetic abnormalities were chromosome 1q21+ and del(13q14). Failure to achieve ≥very good partial response (VGPR) after first-line therapy independently predicted inferior overall survival (OS) (HR = 0.095, 95%CI 0.022-0.421, *p* = 0.002) and shorter median time to next therapy (TTNT) (HR = 0.088, 95%CI 0.024-0.329, *p*<0.001). Intensified regimens combining proteasome inhibitors (PIs)/immunomodulatory drugs (IMiDs) with venetoclax (Ven) significantly improved ≥VGPR rates, particularly when followed by autologous stem cell transplantation (ASCT), prolonged median OS.

**Conclusion:**

Despite novel agents, pPCL maintains poor prognosis, with treatment failure strongly associated with suboptimal first-line response. Therapeutic intensification through PIs/IMiDs with Ven (guided by t(11;14)) and ASCT consolidation may enhance depth of remission and survival outcomes.

## Introduction

Plasma cell leukemia (PCL), a rare and aggressive hematologic malignancy, is defined by the presence of clonal circulating plasma cells (CPCs) in peripheral blood. Primary PCL (pPCL) arises *de novo* without prior multiple myeloma (MM), whereas secondary PCL (sPCL) evolves from pre-existing MM and constitutes 60% of all PCL (1–3). Historically, the International Myeloma Working Group (IMWG) required ≥20% CPCs for pPCL diagnosis (4). However, accumulating evidence indicates that even patients with 5-19% CPCs exhibit comparable poor prognosis to classical pPCL (5–7), prompting the IMWG in 2021 to revise the diagnostic threshold to ≥5% CPCs (8).

This reclassification poses a critical therapeutic dilemma, as many patients now meeting the updated pPCL criteria were previously classified as MM and received conventional therapies that are suboptimal for this high-risk entity, potentially contributing to first-line treatment failure (9–11). While modern induction regimens for pPCL mirror MM strategies–combining proteasome inhibitors (PIs), immunomodulatory drugs (IMiDs), and autologous stem cell transplantation (ASCT)–the outcomes remain dismal, with median overall survival (OS) rarely exceeding 12 months in the novel agent era (12, 13). Notably, the therapeutic potential of emerging agents (e.g., BCL2 inhibitors) in pPCL remains underexplored, largely due to disease rarity and reliance on retrospective data (14–18).

To address this knowledge gap, we analyzed 46 pPCL patients (diagnosed per 2021 IMWG criteria) treated at Nanjing Drum Tower Hospital (2014-2025) with PI/IMiD-based regimens. This retrospective study aimed to delineate their clinical characteristics and analyze the treatments and prognostic determinants.

## Patients and methods

### Patients

This was a retrospective analysis of 46 pPCL patients diagnosed between March 2014 and April 2025 at Drum Tower Hospital, China. All patients were diagnosed by the 2021 modified criteria established by the IMWG. The sPCL patients were excluded from study. The following clinical characteristics were included: age, gender, hemoglobin level, white blood cell level, platelet level, β2-microglobulin (β2-MG) level, lactate dehydrogenase (LDH) level, creatinine (CRE) level, calcium level, albumin level, CPCs, presence of extramedullary disease (EMM), paraprotein isotype, presence of cytogenetic abnormalities characterized by karyotype and fluorescence *in situ* hybridization (FISH), and MM stages as defined by Durie-Salmon (DS) system, the International Scoring System (ISS) system, the Revised-International Staging System (R-ISS) system, and the Second Revision of the International Staging System (R2-ISS). Ethical approval for our investigation was obtained from Drum Tower Hospital’s ethics committee, and informed consents were duly acquired from each participant.

### Flow cytometry analysis

Immunophenotyping was performed on bone marrow and peripheral blood samples using a Beckman Coulter NAVIOS flow cytometer (Beckman Coulter, Miami, USA). Cells were stained with fluorochrome-conjugated antibodies against CD38, CD138, CD56, CD117, CD19, and kappa/lambda light chains (all from Beckman Coulter Immunotech, Marseille, France). A well-established positivity threshold of ≥20% of the gated plasma cell population was applied for all markers. Results are reported as the percentage of positive cells within the identified CD38^+^/CD138^+^ malignant plasma cell cluster.

### Assessment of treatment efficacy

All patients in the study underwent one or more cycles of treatment, and their disease status were assessed after every cycle of treatment, in accordance with the IMWG criteria (4). Therapeutic outcomes were classified as complete response (CR), very good partial response (VGPR), partial response (PR), stable disease (SD) and progressive disease (PD). The overall response rate (ORR) was defined as PR or better. The OS was calculated from the date of diagnosis to the day of last follow-up or death. The time to next therapy (TTNT) was measured from the day of diagnosis to the day of initiating the next systemic therapy due to progression or a documented relapse of disease.

### Immunohistochemistry

The formalin-fixed, paraffin-embedded sections were used for immunohistochemistry (IHC) with the CD38 (ZM-0422, ZSGB-BIO, China) and BCL2 (ZA-0536, ZSGB-BIO, China) antibodies, according to the manufacturer’s protocol. Aggregates of plasma cells were assessed for CD38 and BCL2 in sequential slides. Without prior knowledge of clinical outcomes, two pathologists independently graded the BCL2 expression as follows: 0 level, <10% of tumor cells with no staining; 1+ level, ≥10% tumor cells with weak staining intensity; 2+ level, ≥10% tumor cells with moderate staining intensity; and 3+ level, ≥10% tumor cells with strong staining intensity, with a predefined BCL2^high^ cut-off at 3+ levels (**Supplementary Figure S2**).

### Statistical analysis

All statistical analyses were conducted using SPSS software (version 23.0). The Chi-squared or Fisher tests were employed to identify relationships between patients’ clinical characteristics and treatment outcomes. OS and TTNT were evaluated using the Kaplan-Meier analysis, and comparisons were performed by the log-rank test. Furthermore, the univariate and multivariate analyses were conducted using the Cox proportional hazards model to evaluate the influence of various prognostic factors and their ability to predict OS and TTNT. The *p*<0.05 was used as the cutoff for statistical significance in all analyses.

## Results

### Clinical and laboratory manifestations

The baseline characteristics of the 46 pPCL patients are summarized in **Table 1**. The median age was 62.5 years (range: 43-79), with a male predominance (56.5%, 26/46). Anemia defined by hemoglobin <85g/L was diagnosed in 82.6% of this cohort. Elevated levels of serum LDH, creatinine, calcium, and β2-MG were 52.2%, 41.3%, 45.7%, and 95.7% of this cohort, respectively. The percentage of pPCL patients who had EMM was 39.1%. The EMM presentations were categorized as follows: 9 patients (50.0%) had isolated extramedullary bone involvement (EMB), 8 patients (44.4%) had isolated extraosseous involvement (EME), and 1 patient (5.6%) presented with combined EMB and EME manifestations. The distribution of paraprotein isotype among this cohort was as follows: immunoglobulin (Ig) G, 18 (39.1%); IgA, 7 (15.2%); IgD, 5 (10.9%); IgM, 2 (4.3%); and light chain 14 (30.4%). Immunophenotypic profiles revealed consistent plasma cell lineage commitment: Bone marrow: CD38^+^ (100%, 46/46), CD138^+^ (100%, 46/46), CD19^-^ (100%, 41/41), CD56^+^ (40.5%, 17/42), CD117^-^ (70%, 28/40);Peripheral blood: CD38^+^ (100%, 29/29), CD138^+^ (100%, 29/29), CD19^-^ (100%, 29/29), CD56^+^ (37.9%, 11/29), CD117^-^ (82.8%, 24/29). As to cytogenetic abnormalities by conventional karyotyping, the detection rates of complex karyotype were 48.7%. As to cytogenetic abnormalities by FISH testing, the detection rates of 1q21+, del(17p), del (13q14), t(4;14), t(11;14), t(14;16), and t(14;20) were 70.5%, 27.9%, 64.7%, 20.6%, 37.8%, 11.8%, and 6.1% in this cohort, respectively. The 1q21+ and del(13q14) are the most common cytogenetic abnormalities in our cohort. Based on the DS, ISS, and R-ISS staging systems, 46 pPCL patients were stratified as follows: Stage I & II: 2.2%, 19.6%, and 34.8%; Stage III: 97.8%, 80.4% and 65.2%. The proportions of the advanced stage were more frequent in the pPCL (*p*<0.05; **Table 1**). To contextualize our cohort within contemporary risk frameworks, we also applied the R2-ISS. Among patients with sufficient data (n=43), the distribution was: I&II: 11.6%, III&IV: 88.4%, underscoring the prevalence of high-risk disease in this cohort (*p*<0.001; **Supplementary Table S2**). In a *post-hoc* analysis comparing patients with 5-19% CPCs (n=30) to those with ≥20% CPCs (n=16), we found no significant differences in key high-risk features such as the frequency of EMM (36.7% vs 43.8%, *p* = 0.754), elevated LDH (43.3% vs 68.8%, *p* = 0.129), del(17p) (25.9% vs 31.3%, *p* = 0.737), or 1q21+ (67.9% vs 75.0%, *p* = 0.738) (**Supplementary Table S3**).

**Table 1:** Main clinical and laboratory manifestations of 46 pPCL patients.

Clinical characteristics		P-value
Sex, male [n (%)]	26/46 (56.5)	–
Age (years), M (range)	62.5 (43.0-79.0)	–
White blood cell count (109/L), M (range)	5.95 (1.00-33.90)	–
Hemoglobin<85g/L [n (%)]	38/46 (82.6)	–
Platelets<100×109/L [n (%)]	28/46 (60.9)	–
CPCs [n (%)]		
5-19%	30/46 (65.2)	
≥20%	16/46 (34.8)	
Elevated LDH [n (%)]	24/46 (52.2)	–
Elevated creatine in serum (>177umol/L) [n (%)]	19/46 (41.3)	–
Elevated albumin-corrected calcium in serum [n (%)]	21/46 (45.7)	–
Elevated β2-MG in serum ( ≥3.5mg/L) [n (%)]	44/46 (95.7)	–
Albumin <35g/L [n (%)]	23/46 (50.0)	–
Extramedullary disease [n (%)]	18/46 (39.1)	–
Paraprotein isotype [n (%)]		
IgG	18/46 (39.1)	
IgA	7/46 (15.2)	
IgD	5/46 (10.9)	
IgM	2/46 (4.3)	
Light chain only	14/46 (30.4)	
Phenotype expression [n (%)] (BM)		
CD138 positive	46/46(100.0)	
CD38 positive	46/46 (100.0)	
CD56 positive	17/42 (40.5)	
CD117 negative	28/40 (70.0)	
CD19 negative	41/41 (100.0)	
Kappa positive	25/46 (54.3)	
Lambda positive	21/46 (45.7)	
Phenotype expression [n (%)] (PB)		
CD138 positive	29/29 (100.0)	
CD38 positive	29/29 (100.0)	
CD56 positive	11/29 (37.9)	
CD117 negative	24/29 (82.8)	
CD19 negative	29/29 (100.0)	
Kappa positive	17/29 (58.6)	
Lambda positive	12/29 (41.4)	
Complex karyotype [n (%)]	19/39 (48.7)	–
Cytogenetic abnormality [n (%)]		
1q21+	31/44 (70.5)	
del (17p)	12/43 (27.9)	
del (13q14)	11/17 (64.7)	
t (4;14)	7/34 (20.6)	
t (11;14)	14/37 (37.8)	
t (14;16)	4/34 (11.8)	
t (14;20)	2/33 (6.1)	
Durie-Salmon staging system [n (%)]		**0.022**
I&II	1/46 (2.2)	
III	45/46 (97.8)	
International staging system [n (%)]		**<0.001**
I&II	9/46 (19.6)	
III	37/46 (80.4)	
Revised international staging system [n (%)]		**<0.001**
I&II	16/46 (34.8)	
III	30/46 (65.2)	

Bold values indicate that the P value is less than 0.05 and can be adjusted to be consistent with other values.

### Prognostic factors

**Figure 1:**
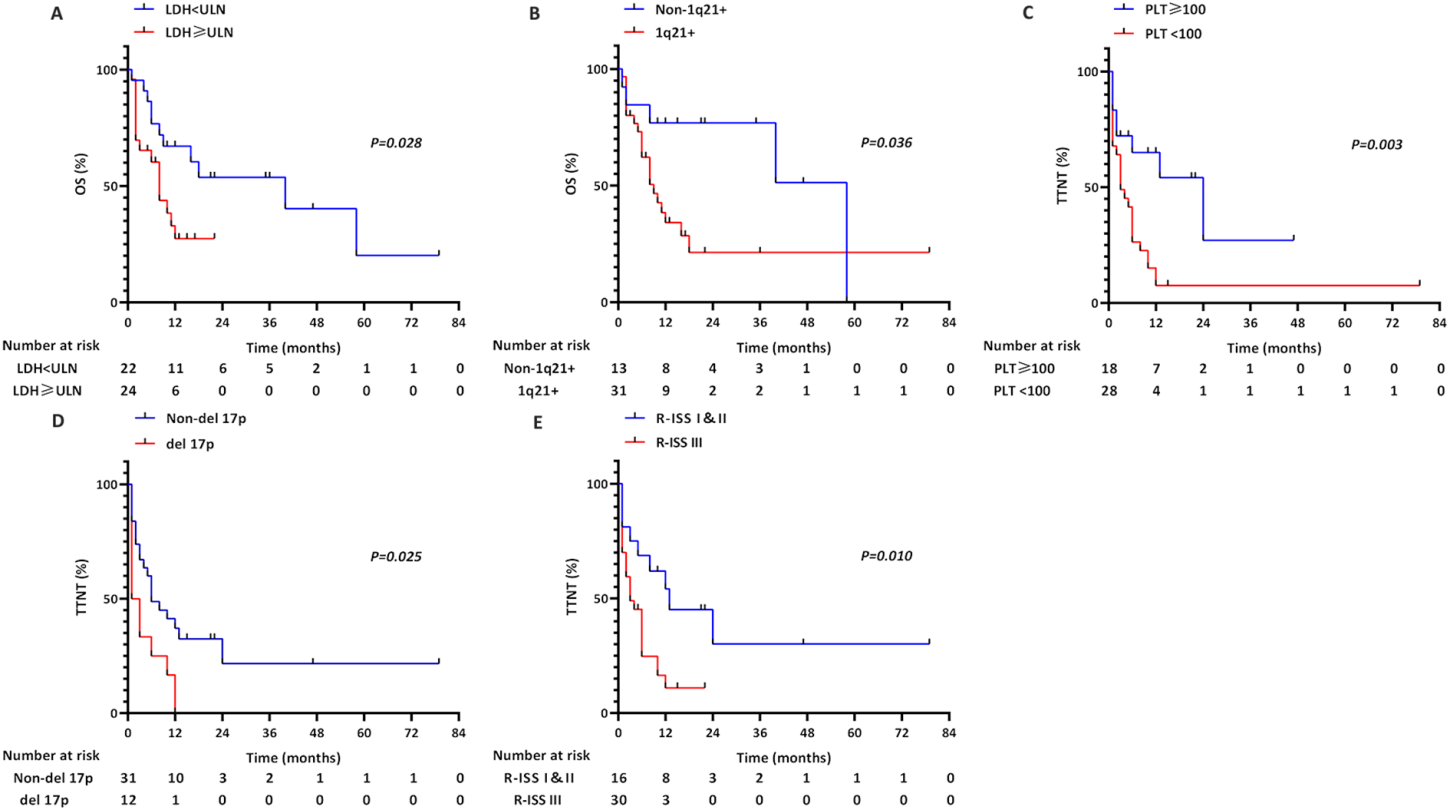
TTNT and OS in pPCL patients stratified by: **(A)** Serum LDH levels; **(B)** 1q21+ status; **(C)** Platelet (PLT) counts; **(D)** del(17p) status; **(E)** R-ISS stage.

With a median follow-up of 21 months (range 1-79), the cohort demonstrated rapid disease progression, reflected by a median TTNT of 6 months and OS of 12 months. Univariate analysis showed that Platelets<100×10^9^/L, LDH>ULN, 1q21+, del(17p), R-ISS III, and ≥VGPR/first-line therapy have impacts on OS and TTNT, according to the Cox proportional hazards model (**Table 2**). Subsequently, multivariate analysis showed that achieve ≥VGPR after first-line therapy emerged as the sole independent predictor of OS (HR = 0.095, 95%CI 0.022-0.421, *p* = 0.002) and TTNT (HR = 0.088, 95%CI 0.024-0.329, *p*<0.001) in this cohort (**Table 3**). Furthermore, survival curves were plotted using the Kaplan-Meier method, with comparison using the log-rank test. When stratifying by LDH, the median OS was 8 months for those with LDH>ULN compared with 40 months for those with low LDH levels (*p* = 0.028; **Figure 1A**). When stratifying by 1q21+, the median OS was 9 months for those with 1q21+ compared with 58 months for those with Non-1q21+ (*p* = 0.036; **Figure 1B**). When stratifying by Platelets, the median TTNT was 3 months for those with Platelets<100×10^9^/L compared with 24 months for those with Platelets>100×10^9^/L (*p* = 0.003; **Figure 1C**). When stratifying by del(17p), the median TTNT was 1 months for those with del(17p) compared with 6 months for those with non-del(17p) (*p* = 0.025; **Figure 1D**). When stratifying by R-ISS, the median TTNT was 3 months for those with R-ISS III compared with 13 months for those with R-ISS I&II (*p* = 0.01; **Figure 1E**).

**Table 2:** Univariate analysis of clinical characteristics predictive of OS and TTNT.

Clinical characteristics	OS		TTNT	
	P-value	HR (95%CI)	P-value	HR (95%CI)
Sex, Female	0.549	0.779 (0.344-1.764)	0.859	1.066 (0.528-2.152)
Age≥65 years	0.417	1.397 (0.623-3.136)	0.323	1.427 (0.705-2.888)
White blood cell count>10×109/L	0.878	1.088 (0.368-3.221)	0.643	0.798 (0.307-2.074)
Hemoglobin<85g/L	0.845	0.906 (0.336-2.443)	0.480	1.411 (0.543-3.669)
Platelets<100×109/L	0.107	2.042 (0.857-4.870)	**0.010**	2.915 (1.297-6.555)
Albumin<35g/L	0.464	0.744 (0.337-1.641)	0.883	1.053 (0.531-2.087)
LDH>ULN	**0.038**	2.461 (1.049-5.773)	0.182	1.621 (0.798-3.293)
Extramedullary disease	0.232	1.619 (0.735-3.563)	0.292	1.447 (0.728-2.873)
CPCs≥20%	0.468	1.346 (0.604-3.001)	0.733	0.882 (0.427-1.821)
Complex karyotype	0.487	1.370 (0.564-3.329)	0.165	1.737 (0.797-3.788)
CD56 positive	0.203	1.777 (0.734-4.299)	0.072	1.993 (0.941-4.220)
CD117 negative	0.663	0.806 (0.305-2.126)	0.941	1.032 (0.450-2.363)
t (4;14)	0.524	1.445 (0.466-4.485)	0.187	1.893 (0.733-4.887)
t (14;16)	0.665	1.318 (0.378-4.594)	0.388	1.611 (0.545-4.766)
t (14;20)	0.477	0.044 (0.000-237.950)	0.620	0.596 (0.077-4.614)
t (11;14)	0.864	0.922 (0.362-2.344)	0.334	0.660 (0.283-1.535)
1q21+	**0.049**	2.736 (1.003-7.464)	0.062	2.253 (0.961-5.286)
del 17p	0.128	1.983 (0.820-4.794)	**0.045**	2.178 (1.019-4.654)
del (13q14)	0.407	1.757 (0.464-6.650)	0.254	2.150 (0.577-8.017)
ISS III	0.948	1.031 (0.410-2.594)	0.373	1.502 (0.614-3.676)
R-ISS III	0.070	2.385 (0.932-6.100)	**0.022**	2.626 (1.152-5.988)
≥VGPR/first-line therapy	**0.002**	0.095 (0.022-0.410)	**<0.001**	0.114 (0.038-0.341)

Bold values indicate that the P value is less than 0.05 and can be adjusted to be consistent with other values.

**Table 3:** Multivariate analysis of clinical characteristics predictive of OS and TTNT.

Clinical characteristics	OS		TTNT	
	P-value	HR (95%CI)	P-value	HR (95%CI)
Platelets<100×109/L	–	–	0.062	2.267 (0.960-5.354)
LDH>ULN	0.102	2.139 (0.860-5.315)	–	–
1q21+	0.247	1.925 (0.636-5.827)	–	–
del 17p	–	–	0.163	1.820 (0.784-4.225)
R-ISS III	–	–	0.225	1.871 (0.680-5.150)
≥VGPR/first-line therapy	**0.002**	0.095 (0.022-0.421)	**<0.001**	0.088 (0.024-0.329)

Bold values indicate that the P value is less than 0.05 and can be adjusted to be consistent with other values.

**Figure 2:**
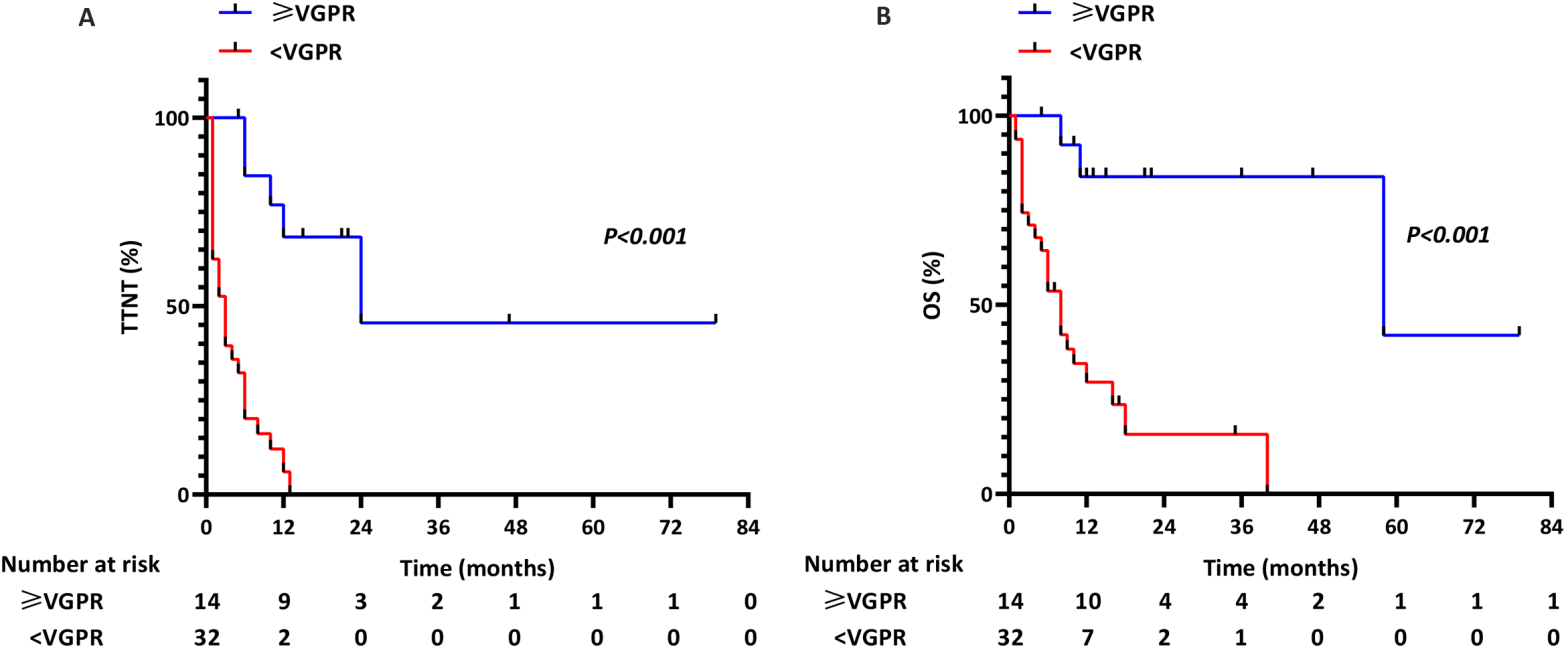
TTNT and OS in pPCL patients stratified by achievement of ≥VGPR with first-line therapy: **(A)** TTNT; **(B)** OS.

The TTNT for patients who experienced ≥VGPR after first-line therapy was 24 months compared with 3 months in patients whose response was poorer efficacy (*p*<0.001; **Figure 2A**). Similarly, the OS for patients who experienced ≥VGPR after first-line therapy was 58 months compared with 8 months in patients whose response was poorer efficacy (*p*<0.001; **Figure 2B**). The 58-month OS in ≥VGPR achievers challenges historical perceptions of pPCL prognosis, suggesting depth of response as a modifiable survival determinant.

### First-line treatment regimens and outcome

Initial therapies were heterogeneous, as detailed in **Supplementary Table S1**. All 46 pPCL patients received upfront PI- or IMiD-based induction regimens evaluated per IMWG criteria (**Table 4**): PI-based therapies (46/46): Bortezomib (40/46), Ixazomib/Carfilzomib (6/46); IMiD-based therapies (33/46): Thalidomide (6/33), Lenalidomide/Pomalidomide (27/33). No difference in ≥VGPR rates existed between early- and new-generation PIs/IMiDs, though new-generation drugs conferred a survival advantage. Significantly higher ORR occurred with Carfilzomib (100%) and Pomalidomide (100%) versus other agents.

**Table 4:** Summary of best response according to different generations of PIs and IMiDs.

Therapy	Treated patients, n	CR, n	VGPR, n	PR, n	SD, n	PD, n	≥VGPR, %	P-value	ORR, %	P-value	Median OS	P-value	Median TTNT	P-value
PI	46	6	8	16	10	6	30	1.000	65	0.084	12.0	0.799	6.0	0.241
Bortezomib	40	6	7	12	10	5	33		63		12.0		6.0	
Carfilzomib	5	0	1	4	0	0	20		100		8.0		6.0	
Ixazomib	1	0	0	0	0	1	0		0		9.0		1.0	
IMiD	33	6	8	12	4	3	42	0.643	79	0.811	16.0	0.845	8.0	0.662
Thalidomide	6	2	0	3	1	0	33		83		16.0		8.0	
Lenalidomide	23	4	7	6	3	3	48		74		12.0		10.0	
Pomalidomide	4	0	1	3	0	0	25		100		8.0		6.0	

**Figure 3:**
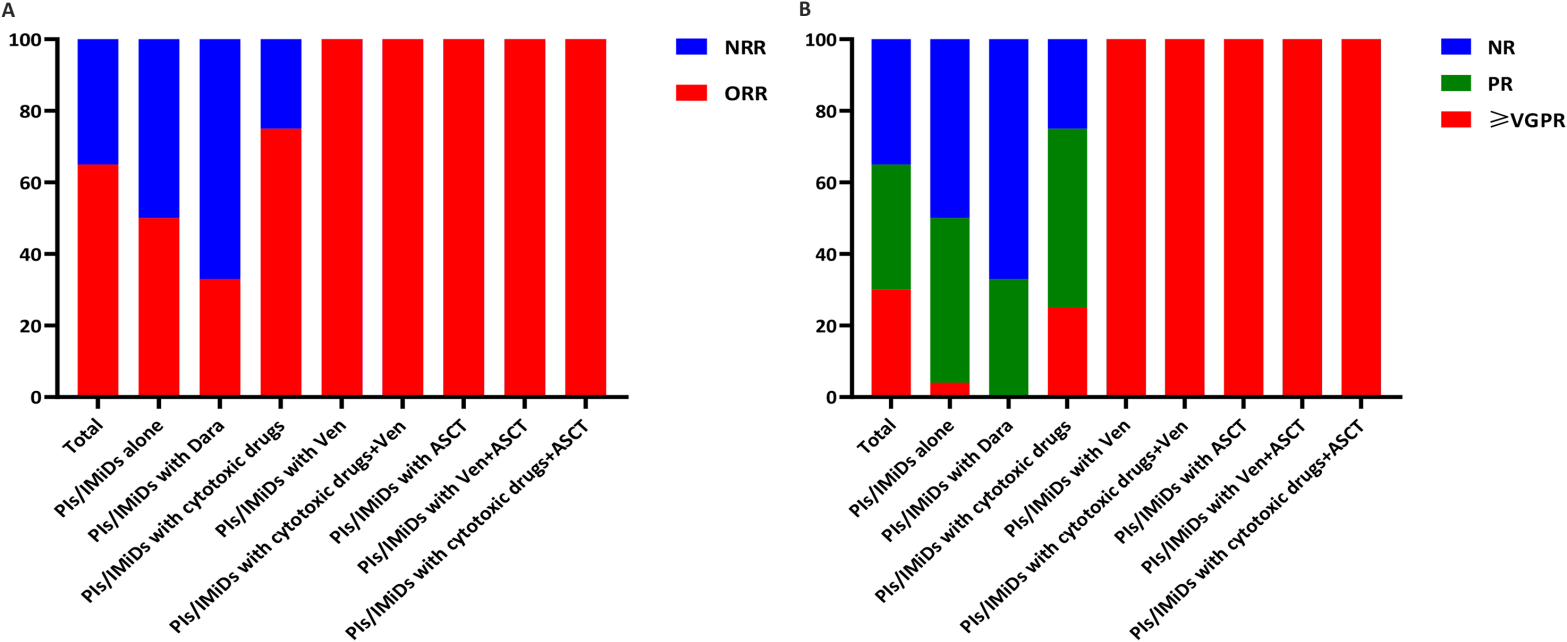
Hematologic response rates to first-line therapy: **(A)** ORR; **(B)** ≥VGPR rate.

Although no consensus exists on optimal induction regimens for pPCL, current guidelines recommend PI- or IMiD-based therapies as the backbone (15, 16). Building on evidence that PI/IMiD combinations outperform monotherapy in response rates (11), our cohort analysis suggests that dual-agent regimens may represent a preferred first-line strategy. To explore novel agents in pPCL, we stratified patients into eight treatment groups (data available for all 46 patients). The ORR was 65%, with ≥VGPR achieved in 30% of patients (**Table 5**). Strikingly, regimens incorporating Ven or ASCT demonstrated superior efficacy (ORR: 100%, *p*<0.05, **Figure 3A**; ≥VGPR: 100%, *p*<0.05, **Figure 3B**).

**Table 5:** Summary of best response according to different treatments.

Therapy	Treated patients, n	CR, n	VGPR, n	PR, n	SD, n	PD, n	≥VGPR, %	*P*-value	ORR, %	*P*-value	Median OS	*P*-value	Median TTNT	*P*-value
Group 1														
PI or/and IMiD alone	24	0	1	11	8	4	4	<0.001	50	0.008	6.0	<0.001	3.0	<0.001
PI or/and IMiD with Dara	3	0	0	1	1	1	0		33		9.0		1.0	
PI or/and IMiD with cytotoxic drugs	8	2	0	4	1	1	25		75		18.0		6.0	
Group 2														
PI or/and IMiD with Ven	3	1	2	0	0	0	100		100		–		–	
PI or/and IMiD with cytotoxic drugs+Ven	1	0	1	0	0	0	100		100		–		6.0	
PI or/and IMiD with ASCT	4	1	3	0	0	0	100		100		–		–	
PI or/and IMiD with Ven+ASCT	2	1	1	0	0	0	100		100		11.0		10.0	
PI or/and IMiD with cytotoxic drugs+ASCT	1	1	0	0	0	0	100		100		–		–	
Total	46	6	8	16	10	6	30		65		12.0		6.0	

### Characteristics of patients with deeper response and longer survival

**Figure 4:**
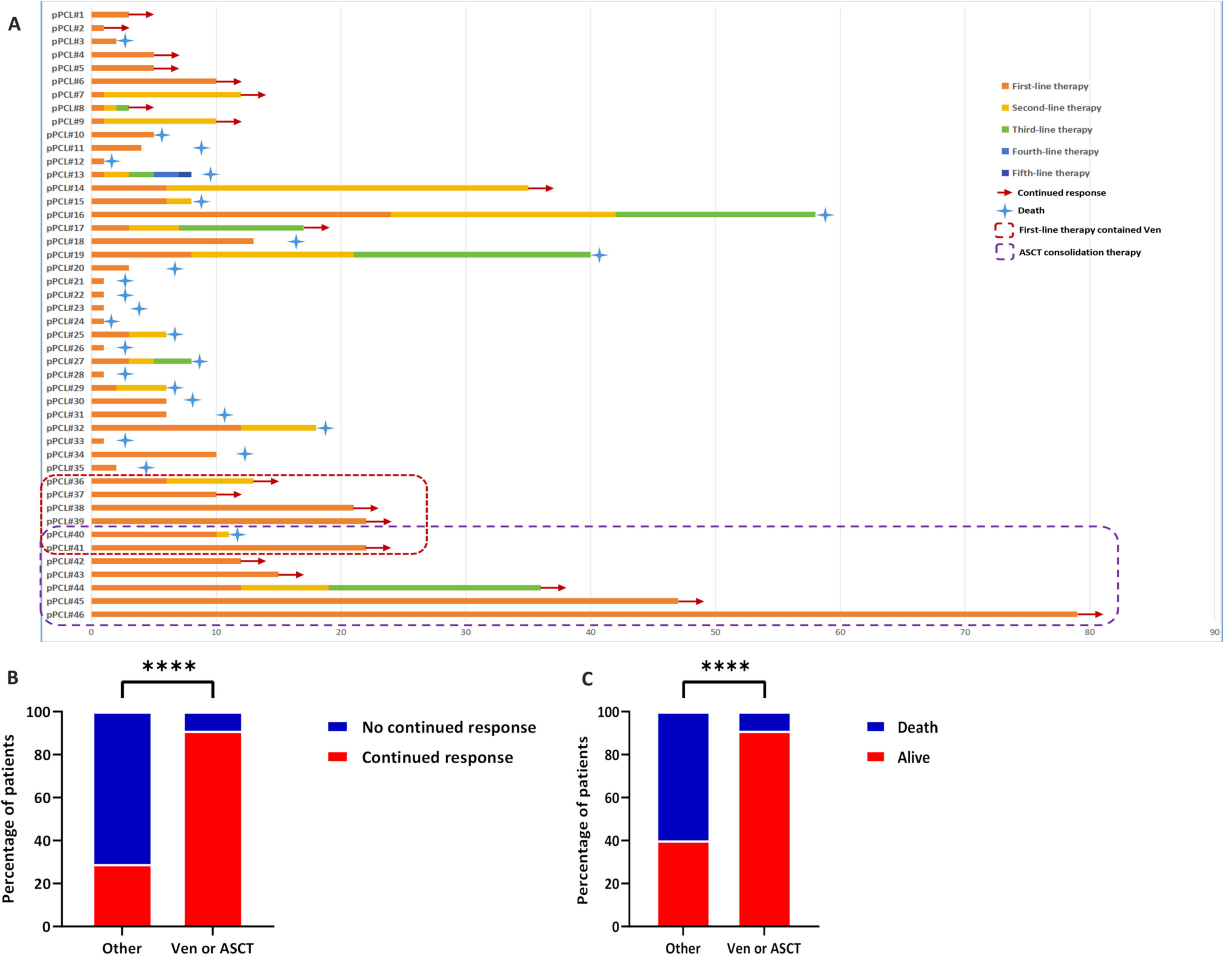
Treatment sequencing and outcomes: **(A)** Swimmer plot of therapy duration across sequential lines in 46 pPCL patients; **(B)** Sustained response rates: PI/IMiD + Ven/ASCT vs. other regimens; **(C)** First-year mortality: PI/IMiD + Ven/ASCT vs. other regimens.

**Figure 5:**
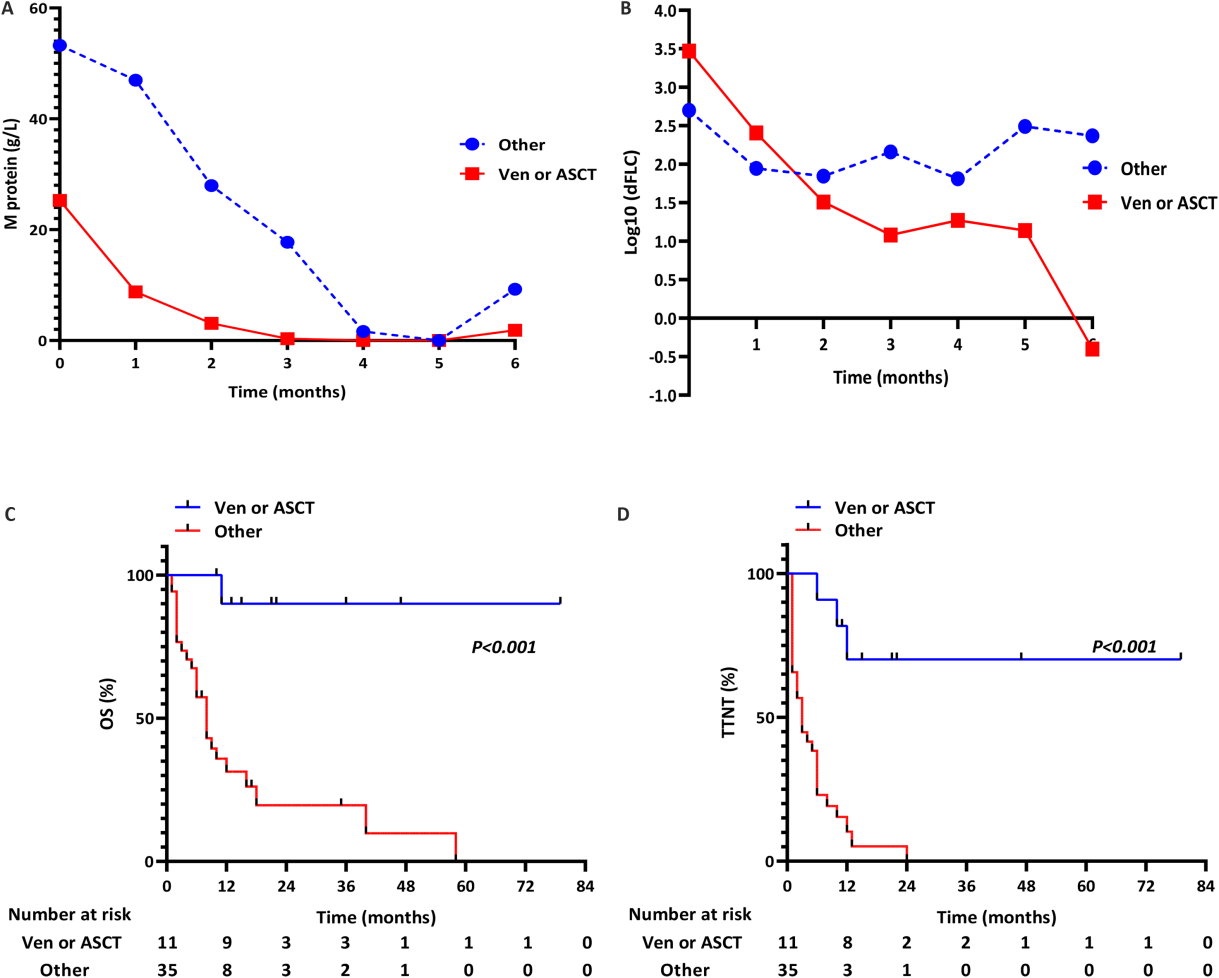
Response kinetics and survival patterns: **(A, B)** Serial quantification of: M-protein levels and Free light chain levels; **(C, D)** Survival outcomes by treatment: OS and TTNT.

To characterize pPCL patients achieving deeper responses, we stratified them by treatment regimen (**Table 5**). Sequential treatment data were available for all 46 patients (**Figure 4A**): Group 1 (PIs/IMiDs + others): pPCL#1-35; Group 2 (PIs/IMiDs + Ven/ASCT): pPCL#36-46. Patients receiving PIs/IMiDs + Ven/ASCT showed significantly higher sustained response rates (*p*<0.001; **Figure 4B**) and reduced early mortality (*p*<0.001; **Figure 4C**). Response durations (assessed by M-protein and free light chain) are shown in **Figures 5A, B**. For survival analysis, both OS and TTNT were significantly longer in Group 2 versus Group 1 (OS: *p*<0.001, **Figure 5C**; TTNT: *p*<0.001, **Figure 5D**). These findings suggest that incorporating Ven or ASCT after PI/IMiD-based induction correlates with deeper hematologic remission and prolonged survival in pPCL.

### Venetoclax in upfront induction therapy for pPCL with t(11;14)

The cytogenetic landscape of pPCL provides a rationale for Ven-based targeted therapy. While t(11;14) present in approximately 65% of pPCL cases is an established predictor of Ven sensitivity (11, 19–24), emerging evidence suggests broader applicability of BCL2 inhibition (25–27). We evaluated BCL2 expression in bone marrow samples using IHC and found universal BCL2 expression across all 33 available patient samples (100% positivity). However, when applying the 3+ cut-off, BCL2 expression showed no significant association with t(11;14) status (*p* = 1.00; **Supplementary Table S4**). Similarly, no significant differences in OS (*p* = 0.272) or TTNT (*p* = 0.530) were observed between BCL2^high^(3+) and BCL2^low^(0/1+/2+) expressors (**Supplementary Figure S2**), though we acknowledge the limited statistical power of these subgroup analyses. In this cohort, there were 6 cases who received Ven-based induction regimens. All 6 cases received Ven as part of their induction therapy, which was subsequently continued as maintenance treatment in responders. The most common regimen combined Ven with IMiDs (lenalidomide or pomalidomide). The Ven dosing was initiated at 400 mg daily during the first 14-days cycle. IMiD therapy was then introduced orally beginning on day 15. Even though only 4 cases (67%) had t(11;14) translocations, all 6 cases (100%) received ≥VGPR/first-line therapy. Notably, two patients without t(11;14) also achieved ≥VGPR but experienced short remission durations with subsequent relapse and death (**Table 6**). This demonstrates Ven’s ability to induce hematologic responses (including VGPR/CR) in BCL2 high expressing pPCL, though remissions were unsustainable in non-t(11;14) cases. Further investigation is needed given reports of Ven’s failure to prevent progression in PCL with or without t(11;14).

**Table 6:** Clinical outcomes with Ven-based induction.

Subgroup	t(11;14)	BCL2^high^/non-t(11;14)
Patients	4 (67%)	2 (33%)
≥VGPR achievement	100%	100% (Cycle 1)
Response durability	Sustained	Short remission→ relapse/death

## Discussion

pPCL demonstrates more aggressive clinical behavior at diagnosis compared to MM. While the IMWG historically required ≥20% CPCs for pPCL diagnosis, accumulating evidence has shown that patients with 5-19% CPCs exhibit similarly poor prognosis as those with classical pPCL. This evidence prompted the IMWG’s 2021 revision of the diagnostic threshold to ≥5% CPCs. Although our study was not initially designed with statistical power for formal comparison between these diagnostic thresholds, we conducted a comprehensive *post-hoc* analysis to address this important clinical question. Our comparison of patients with 5-19% CPCs versus those with ≥20% CPCs revealed no statistically significant differences in key high-risk features, including the frequency of EMM, elevated LDH, del(17p), or 1q21+ abnormalities. Furthermore, clinical outcomes were comparable between the two groups, with similar median OS and TTNT (16.0 vs. 9.0 months, *p* = 0.453; 6.0 vs. 6.0 months, *p* = 0.712; **Supplementary Figure S3**) and comparable rates of achieving ≥VGPR after first-line therapy (26.7% vs. 37.5%; *p* = 0.512, **Supplementary Table S5**). These findings provide real-world validation of the updated 2021 IMWG diagnostic criteria, demonstrating that patients with 5-19% CPCs share similar high-risk characteristics, response depth, and survival outcomes with those meeting the classical ≥20% threshold. Our results thus reinforce the clinical rationale underlying the revised diagnostic benchmark for pPCL.

Due to its rarity, current pPCL evidence derives primarily from small retrospective studies and case reports consistent with our findings. The median age is 63 years, approximately 8 years younger than the median age of MM (28). The pPCL is characterized by anemia, hypercalcemia, renal insufficiency, and bone lesions (4). Notably, light-chain isotype predominance distinguishes pPCL from MM (29). While both entities express CD38^+^/CD138^+^ plasma cell markers, pPCL shows significantly reduced CD56 and CD117 expression versus MM (30). Meanwhile, extramedullary involvement is more frequent in pPCL patients, and could be associated with tumor cells having reduced proportions of adhesion molecules (CD56: bone marrow 40.5% and peripheral blood 37.9%), which impair retention of tumor cells within the bone marrow (31). This study introduces novel perspectives on pPCL risk stratification. Given limited prognostic tools for pPCL, we evaluated established MM models (DS/ISS/R-ISS/R2-ISS) in our cohort. pPCL predominantly presented with advanced-stage disease (Stage III: 65-98% across systems), reflecting greater tumor burden (elevated β2-MG: 95.7%; LDH: 52.2%) and high-risk cytogenetics. Critically, pPCL exhibits distinct cytogenetic profiles versus MM, which frequently have complex and hypodiploid karyotypes. The Chromosome 1q21+ and del(13q14) are the most common in pPCL patients. Chromosome 1q21+, considered adverse cytogenetic abnormalities in MM, are significantly frequent in pPCL (32). Chromosome 1 instability is also common structural abnormality and plays a vital role in the pathogenesis of plasma cell malignancy. UAMS-70 genes, 30% mapping to chromosome 1, are related to disease progression and early disease-related death (33, 34). The del(13q14) has been detected in 30-50% of MM patients, with the majority of abnormalities of chromosome 13 representing monosomy. The incidence of del(13q14) in pPCL is higher than that detected in MM, which could infer that deletions of putative tumor suppressor gene at 13q14.3 both lead to the pathogenesis of pPCL and MM (35, 36).

The prognosis of pPCL remains poor globally, with minimal improvement over the past decade despite novel therapies (PIs, IMiDs, daratumumab, cytotoxic agents, ASCT). Median OS with modern induction remains <1 year (8, 10, 13). Our study therefore aimed to identify prognostic determinants for pPCL survival. Despite this cohort’s limited size, our findings align with published data while offering new insights. Our study, which mainly focused on the characteristics of pPCL patients with deeper response and longer survival, showed that the depth of response to induction therapy was an important prognostic factor. In detail, achieving a VGPR as the best hematologic response to first-line therapy was independent of the presences of platelets, LDH, high-risk cytogenetics, and R-ISS III stage in multivariate analysis. Although it is not possible to conclude whether achieving a VGPR is only reflective of less aggressive tumor progression, the likelihood of ≥VGPR/first-line therapy was at least not related to cytogenetic risk and high tumor burden at diagnosis (reflected by LDH and R-ISS III stage). While ≥VGPR may reflect less aggressive biology, its established benefit in ultra-high-risk myeloma suggests deep hematologic responses are likely essential for pPCL outcomes (37).

Given pPCL’s poor prognosis, achieving ≥VGPR with first-line therapy is critical to mitigate early mortality and relapse. While historical studies show conventional chemotherapy yields low response rates and marginal survival gains in pPCL (38, 39). This finding was also corroborated by our cohort, which showed that the use of cytotoxic drugs was related to low response rates and poor survival. As a result, a more intensive treatment with novel agent induction therapy followed by consolidation is an accepted standard of pPCL management. Frontline standards prioritize PI/IMiD combinations, exemplified by VRd (Bortezomib/Lenalidomide/Dexamethasone), which achieves 70% ORR in pPCL (14, 16, 40, 41). At our institution, all pPCL patients would be considered to receive induction therapy regimens combining PIs and/or IMiDs. Importantly, next-generation PIs and IMiDs were also adopted in our cohort. Although there was no difference in the ≥VGPR rates between the early and new generations of PIs and IMiDs, while such patients did not get obviously survival benefits as those receiving next-generation drugs. However, there were significantly higher rates of ORR with Carfilzomib (100%) or Pomalidomide (100%) vs. other agents. These results suggested that the induction phase could be optimized by incorporating novel agents to improve the treatment responses. Furthermore, an important limitation of our study is the lack of frontline incorporation of anti-CD38 monoclonal antibodies, which have become a cornerstone of modern MM therapy and are increasingly used in pPCL. Daratumumab is covered by medical insurance in the region where our center is situated for the treatment of MM. However, its use in patients with pPCL is not included in the reimbursement scheme, requiring out-of-pocket payments. As a result, the high cost poses a significant financial burden, making the treatment unaffordable for the majority of patients with this condition. Future studies prospectively evaluating quadruplet regimens incorporating anti-CD38 antibodies are urgently needed.

For transplant-eligible pPCL patients, intensive novel-agent induction followed by ASCT consolidation is recommended to deepen responses and improve survival. A registry study of 272 pPCL patients demonstrated significantly improved CR rates post-ASCT (41.2%) and median OS of 25.7 months, which was similar to our findings (42). Other registries confirm ASCT’s feasibility and efficacy in pPCL (15, 43). Meanwhile, the roles of double ASCT and Allogeneic Hematopoietic Stem Cell Transplantation (allo-SCT) in pPCL are still controversial, as several retrospective studies have shown that double ASCT and allo-SCT are not related to a significant survival benefit (44–46). Thus, single ASCT consolidation remains standard for eligible patients given its CR rate improvement and survival advantage.

Ven is a potent oral BH3 mimetic that selectively inhibits the antiapoptotic protein BCL2. By displacing proapoptotic proteins (e.g., BIM) and enabling BAX/BAK activation, Ven triggers the intrinsic apoptosis pathway in tumor cell (47, 48). Research on Ven’s antitumor mechanisms has advanced rapidly, with *in vivo* studies confirming efficacy in MM, particularly in t(11;14) (48). Cytogenetic abnormalities are more universal in pPCL than MM, with t(11,14) occurring frequent in pPCL than MM. While t(11;14) is an established Ven sensitivity biomarker, we identified universal BCL2 expression suggesting broader applicability. Our findings demonstrate that Ven induces rapid hematologic responses (≥VGPR) regardless of t(11;14) status. Furthermore, data from the BELLINI study also shows promising efficacy of Ven in patients with high BCL2 gene expression (49). However, responses in non-t(11;14) patients remain transient despite BCL2^high^ status. Both BCL2^high^/non-t(11;14) patients achieved only short-term remissions before experiencing rapid relapse and death. These outcomes demonstrate that BCL2 IHC positivity alone is insufficient for patient selection due to persistent response heterogeneity. Future research on advanced biomarkers beyond IHC (e.g., BCL2/MCL1 ratios, BH3 profiling) and novel combination regimens for non-t(11;14) pPCL should be prioritized.

## Conclusion

This study confirms that pPCL remains an aggressive malignancy with persistently poor prognosis despite novel therapies. We identify achieving ≥VGPR with first-line therapy as a critical determinant of superior survival. The use of multidrug combinations (Ven-containing PI/IMiD combinations) for induction appears to achieve deep hematologic responses (≥VGPR: 100% in cohort) in pPCL. Furthermore, consolidation with a single ASCT should be incorporated in eligible pPCL patients. In the era of novel agent therapy, PI/IMiD backbone + biomarker-directed Ven (t(11;14) -positive) + ASCT consolidation could maximize depth of response and survival outcomes.

## Data Availability

The raw data supporting the conclusions of this article will be made available by the authors, without undue reservation.

## Supplementary material

Supplementary Figure 1 TTNT and OS in non-1q21+ pPCL patients stratified by treatment: (A) OS; (B) TTNT.

Supplementary Figure 2 BCL2 expression in bone marrow and its prognostic relevance in pPCL: (A) Representative IHC images showing CD38 and BCL2 expression in bone marrow samples; (B) Survival analysis comparing patients with high (3+) versus low (0/1+/2+) BCL2 expression: OS and TTNT.

Supplementary Figure 3 Survival outcomes in pPCL patients stratified by CPCs proportion: (A) OS; (B) TTNT.

Supplementary Table 1 Summary of best response according to different treatments.

Supplementary Table 2 Distribution of pPCL patients according to R2-ISS staging.

Supplementary Table 3 Baseline clinical and laboratory characteristics of the 46 pPCL patients, categorized by CPCs proportion.

Supplementary Table 4 Frequency of high BCL2 expression (BCL2^high)^ in patients with or without t(11;14).

Supplementary Table 5 Rate of achieving ≥VGPR after first-line therapy, stratified by CPCs proportion.
