# Supplementary figures and images for: Survival improvement in primary plasma cell leukemia: a retrospective analysis of novel agent-based regimens and stem cell transplantation
Source: Front Oncol. 2026 Jan 9;15:1727117. doi: 10.3389/fonc.2025.1727117 (PMC12827157; doi:10.3389/fonc.2025.1727117)

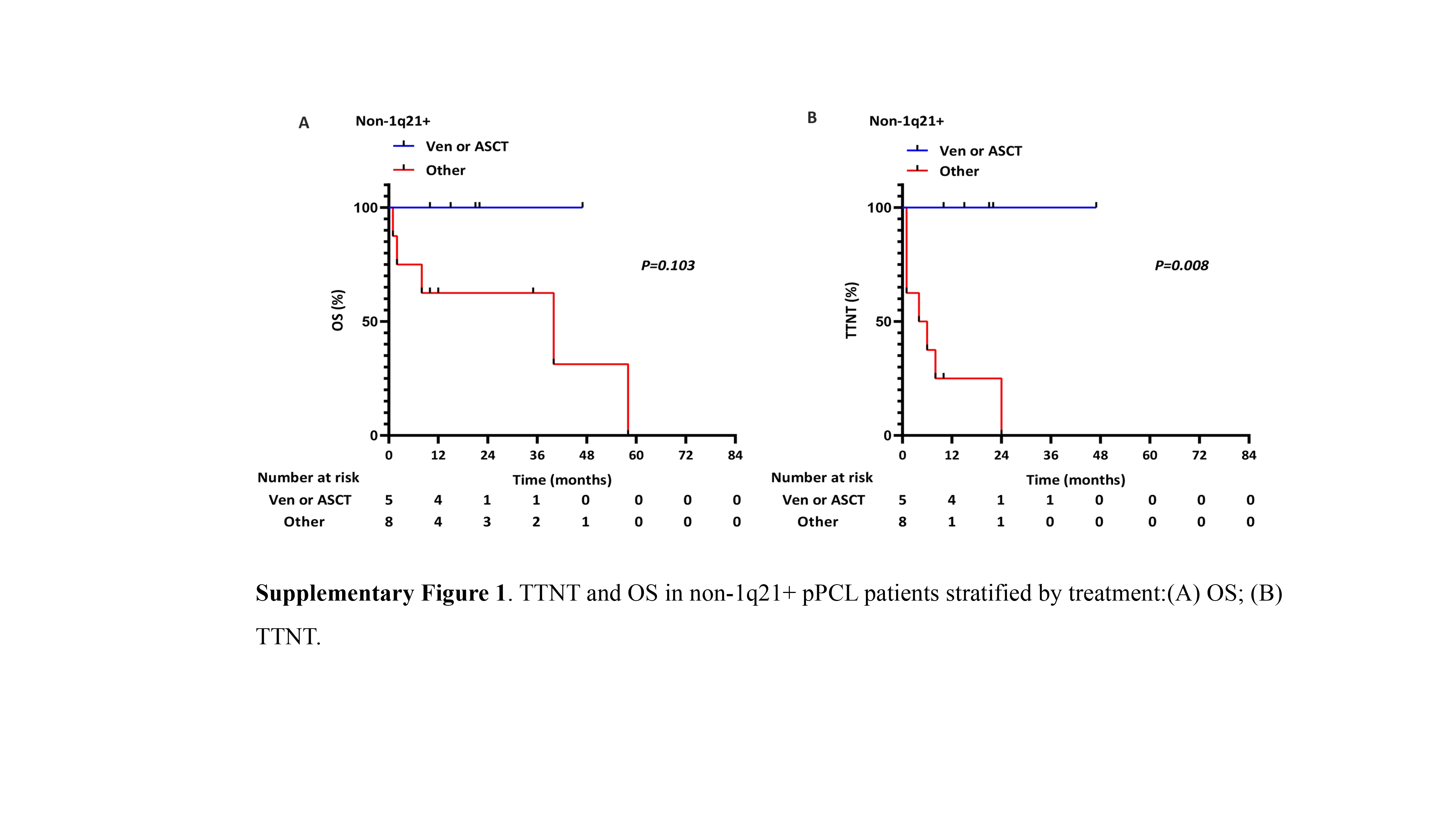

Supplement: Supplementary Figure 1 — TTNT and OS in non-1q21+ pPCL patients stratified by treatment: (A) OS; (B) TTNT. [file Image1.tif]

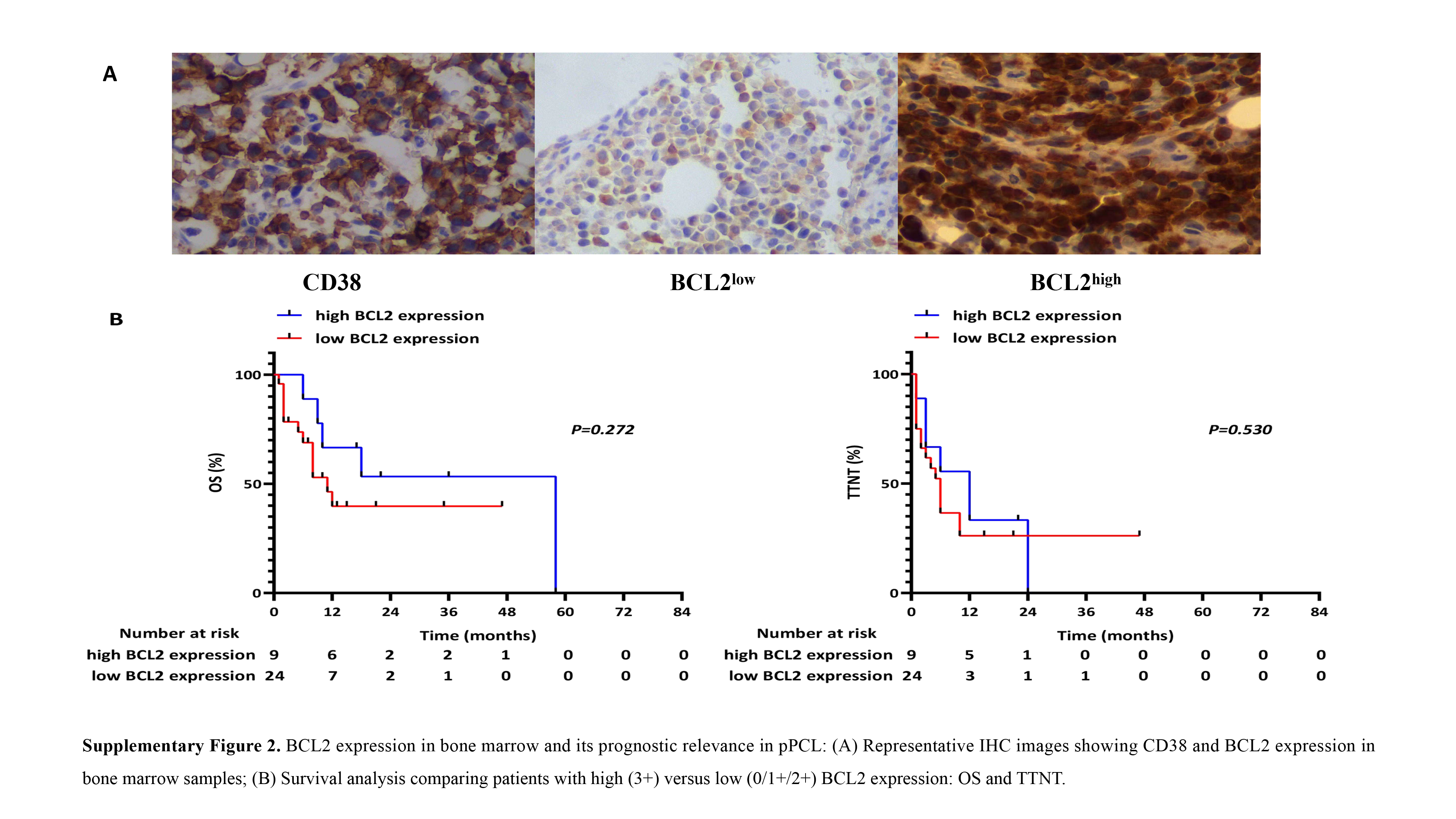

Supplement: Supplementary Figure 2 — BCL2 expression in bone marrow and its prognostic relevance in pPCL: (A) Representative IHC images showing CD38 and BCL2 expression in bone marrow samples; (B) Survival analysis comparing patients with high (3+) versus low (0/1+/2+) BCL2 expression: OS and TTNT. [file Image2.tif]

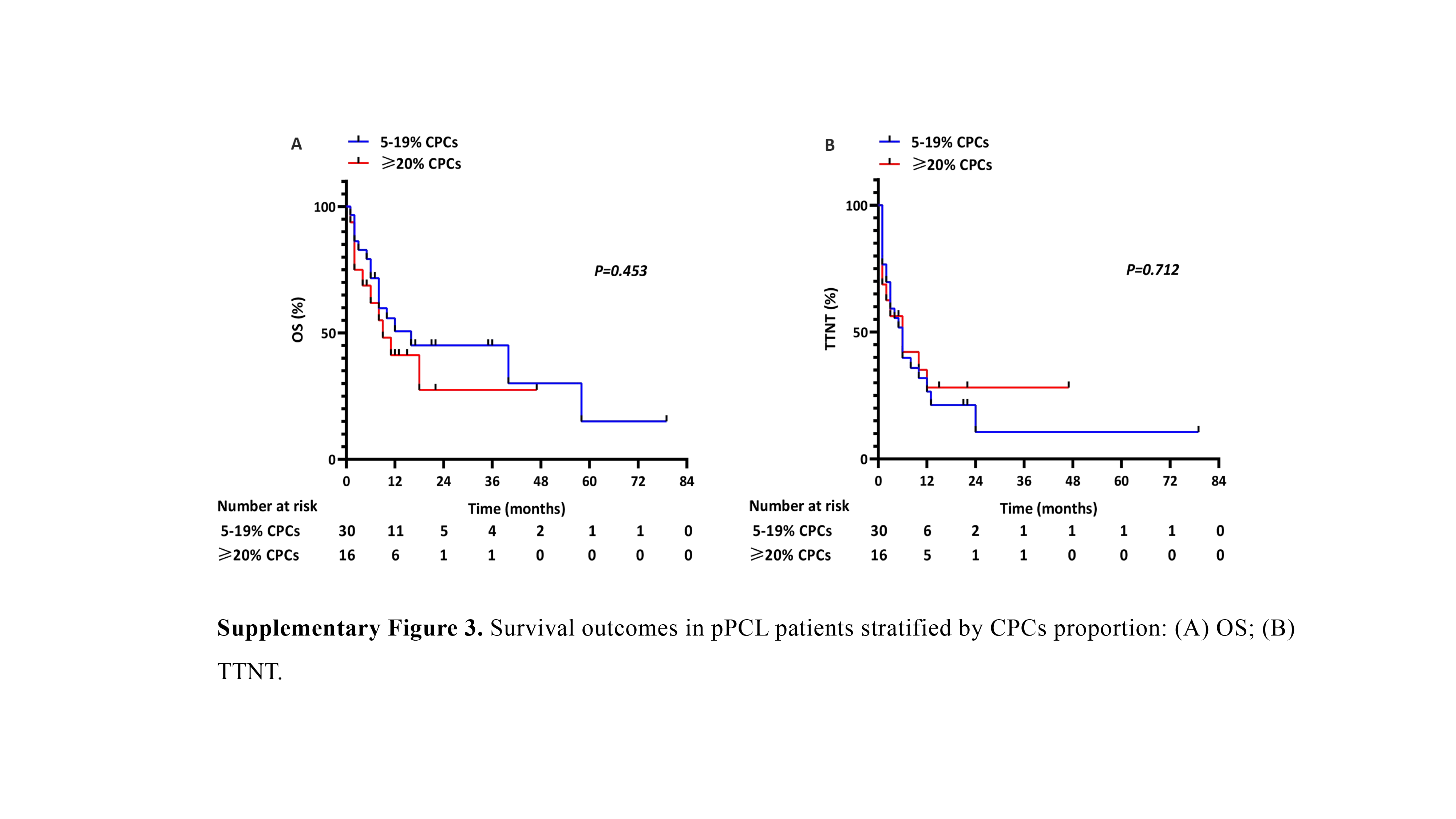

Supplement: Supplementary Figure 3 — Survival outcomes in pPCL patients stratified by CPCs proportion: (A) OS; (B) TTNT. [file Image3.tif]
