# Supplementary material for: Survival improvement in primary plasma cell leukemia: a retrospective analysis of novel agent-based regimens and stem cell transplantation
Source: Front Oncol. 2026 Jan 9;15:1727117. doi: 10.3389/fonc.2025.1727117 (PMC12827157; doi:10.3389/fonc.2025.1727117)
Supplement: Supplementary Table 1 — Summary of best response according to different treatments. [file Table1.docx]

**Supplementary Table 1. Summary of best response according to different treatments.**

| Therapy | Treated patients, n | CR, n | VGPR, n | PR, n | SD, n | PD, n | ≥VGPR, % | ORR, % |
| --- | --- | --- | --- | --- | --- | --- | --- | --- |
| VD | 6 | 0 | 0 | 1 | 4 | 1 | 0 | 17 |
| VTD | 3 | 0 | 0 | 2 | 1 | 0 | 0 | 67 |
| VRD | 12 | 0 | 1 | 5 | 3 | 3 | 8 | 50 |
| VD+cytotoxic drugs | 1 | 0 | 0 | 0 | 1 | 0 | 0 | 0 |
| VTD +HSCT | 1 | 1 | 0 | 0 | 0 | 0 | 100 | 100 |
| VRD+HSCT | 3 | 0 | 3 | 0 | 0 | 0 | 100 | 100 |
| PAD | 3 | 0 | 0 | 2 | 0 | 1 | 0 | 67 |
| DVD | 2 | 0 | 0 | 1 | 1 | 0 | 0 | 50 |
| VRD+Ven | 3 | 1 | 2 | 0 | 0 | 0 | 100 | 100 |
| VRD+Ven+HSCT | 2 | 1 | 1 | 0 | 0 | 0 | 100 | 100 |
| IDD | 1 | 0 | 0 | 0 | 0 | 1 | 0 | 0 |
| KPD | 2 | 0 | 0 | 2 | 0 | 0 | 0 | 100 |
| KRD | 1 | 0 | 0 | 1 | 0 | 0 | 0 | 100 |
| KPD-A-Ven | 1 | 0 | 1 | 0 | 0 | 0 | 100 | 100 |
| KPD-MACE | 1 | 0 | 0 | 1 | 0 | 0 | 0 | 100 |
| VRD-PACE | 1 | 1 | 0 | 0 | 0 | 0 | 100 | 100 |
| VTD+VCD | 2 | 1 | 0 | 1 | 0 | 0 | 50 | 100 |
| VRD-PACE+HSCT | 1 | 1 | 0 | 0 | 0 | 0 | 100 | 100 |
| Total | 46 | 6 | 8 | 16 | 10 | 6 | 30 | 65 |
