# Supplementary material for: Survival improvement in primary plasma cell leukemia: a retrospective analysis of novel agent-based regimens and stem cell transplantation
Source: Front Oncol. 2026 Jan 9;15:1727117. doi: 10.3389/fonc.2025.1727117 (PMC12827157; doi:10.3389/fonc.2025.1727117)
Supplement: Supplementary Table 2 — Distribution of pPCL patients according to R2-ISS staging. [file Table2.docx]

**Supplementary Table 2. Distribution of pPCL patients according to R2-ISS staging.**

| Clinical characteristics |  | *P*-value |
| --- | --- | --- |
| R2-ISS [n (%)] |  | **<0.001** |
| I＆II | 5/43 (11.6) |  |
| III＆IV | 38/43 (88.4) |  |
