# Supplementary material for: Survival improvement in primary plasma cell leukemia: a retrospective analysis of novel agent-based regimens and stem cell transplantation
Source: Front Oncol. 2026 Jan 9;15:1727117. doi: 10.3389/fonc.2025.1727117 (PMC12827157; doi:10.3389/fonc.2025.1727117)
Supplement: Supplementary Table 3 — Baseline clinical and laboratory characteristics of the 46 pPCL patients, categorized by CPCs proportion. [file Table3.docx]

**Supplementary Table 3. Baseline clinical and laboratory characteristics of the 46 pPCL patients, categorized by CPCs proportion.**

| Clinical characteristics | 5-19% CPCs (n=30) | ≥20% CPCs (n=16) | *P*-value |
| --- | --- | --- | --- |
| Sex, male [n (%)] | 16/30 (53.3) | 10/16 (62.5) | 0.756 |
| Age (years), M (range) | 63.5 (43.0-78.0) | 60.5 (46.0-79.0) | 0.239 |
| White blood cell count (10^9^/L), M (range) | 5.15 (1.00-14.40) | 6.65 (1.30-33.90) | **0.037** |
| Hemoglobin<85g/L [n (%)] | 25/30 (83.3) | 13/16 (81.3) | 1.000 |
| Platelets<100×10^9^/L [n (%)] | 17/30 (56.7) | 11/16 (68.8) | 0.533 |
| Elevated LDH [n (%)] | 13/30 (43.3) | 11/16 (68.8) | 0.129 |
| Elevated creatine in serum ( >177umol/L) [n (%)] | 12/30 (40.0) | 7/16 (43.8) | 1.000 |
| Elevated albumin-corrected calcium in serum [n (%)] | 11/30 (36.7) | 10/16 (62.5) | 0.126 |
| Elevated β2-MG in serum ( ≥3.5mg/L) [n (%)] | 28/30 (93.3) | 16/16 (100.0) | 0.536 |
| Albumin <35g/L [n (%)] | 17/30 (56.7) | 6/16 (37.5) | 0.353 |
| Extramedullary disease [n (%)] | 11/30 (36.7) | 7/16 (43.8) | 0.754 |
| Paraprotein isotype [n (%)] |  |  | **0.018** |
| IgG | 16/30 (53.3) | 2/16 (12.5) |  |
| IgA | 4/30 (13.3) | 3/16 (18.8) |  |
| IgD | 3/30 (10.0) | 2/16 (12.5) |  |
| IgM | 0/30 (0.0) | 2/16 (12.5) |  |
| Light chain only | 7/30 (23.3) | 7/16 (43.8) |  |
| Phenotype expression [n (%)] (BM) |  |  |  |
| CD138 positive | 30/30(100.0) | 16/16 (100.0) | - |
| CD38 positive | 30/30(100.0) | 16/16 (100.0) | - |
| CD56 positive | 10/30 (33.3) | 7/16 (43.8) | 0.757 |
| CD117 negative | 18/30 (60.0) | 10/16 (62.5) | 1.000 |
| CD19 negative | 25/25 (100.0) | 16/16 (100.0) | - |
| Kappa positive | 18/30 (60.0) | 7/16 (43.8) | 0.360 |
| Lambda positive | 12/30 (40.0) | 9/16 (56,2) |  |
| Phenotype expression [n (%)] (PB) |  |  |  |
| CD138 positive | 20/20 (100.0) | 9/9 (100.0) | - |
| CD38 positive | 20/20 (100.0) | 9/9 (100.0) | - |
| CD56 positive | 6/20 (30.0) | 5/9 (55.6) | 0.237 |
| CD117 negative | 16/20 (80.0) | 8/9 (88.9) | 1.000 |
| CD19 negative | 20/20 (100.0) | 9/9 (100.0) | - |
| Kappa positive | 13/20 (65.8) | 4/9 (44.4) | 0.422 |
| Lambda positive | 7/20 (35.0) | 5/9 (55.6) |  |
| Complex karyotype [n (%)] | 13/25 (42.0) | 6/15 (40.0) | 0.514 |
| Cytogenetic abnormality [n (%)] |  |  | - |
| 1q21+ | 19/28 (67.9) | 12/16 (75.0) | 0.738 |
| del (17p) | 7/27 (25.9) | 5/16 (31.3) | 0.737 |
| del (13q14) | 6/11 (54.5) | 5/6 (83.3) | 0.333 |
| t (4;14) | 4/21 (19.0) | 3/13 (23.0) | 1.000 |
| t (11;14) | 8/23 (34.8) | 6/14 (42.9) | 0.732 |
| t (14;16) | 1/21 (4.8) | 3/13 (23.1) | 0.274 |
| t (14;20) | 1/20 (5.0) | 1/13 (7.7) | 1.000 |
| Durie-Salmon staging system [n (%)] |  |  | 0.348 |
| I＆II | 0/30 (0.0) | 1/16 (6.3) |  |
| III | 30/30 (100.0) | 15/16 (93.8) |  |
| International staging system [n (%)] |  |  | 0.463 |
| I＆II | 7/30 (23.3) | 2/16 (12.5) |  |
| III | 23/30 (76.7) | 14/16 (87.5) |  |
| Revised international staging system [n (%)] |  |  | 0.117 |
| I＆II | 13/30 (43.3) | 3/16 (18.8) |  |
| III | 17/30 (56.7) | 13/16 (81.3) |  |
