# Supplementary material for: Survival improvement in primary plasma cell leukemia: a retrospective analysis of novel agent-based regimens and stem cell transplantation
Source: Front Oncol. 2026 Jan 9;15:1727117. doi: 10.3389/fonc.2025.1727117 (PMC12827157; doi:10.3389/fonc.2025.1727117)
Supplement: Supplementary Table 4 — Frequency of high BCL2 expression (BCL2high) in patients with or without t(11;14). [file Table4.docx]

**Supplementary Table 4. Frequency of high BCL2 expression (BCL2^high^) in patients with or without t(11;14).**

|  | t(11;14) (n=11) | non-t(11;14) (n=18) | *P*-value |
| --- | --- | --- | --- |
| BCL2^high^ | 3/11 (27.3) | 6/18 (33.3) | 1.000 |
