# Supplementary material for: Survival improvement in primary plasma cell leukemia: a retrospective analysis of novel agent-based regimens and stem cell transplantation
Source: Front Oncol. 2026 Jan 9;15:1727117. doi: 10.3389/fonc.2025.1727117 (PMC12827157; doi:10.3389/fonc.2025.1727117)
Supplement: Supplementary Table 5 — Rate of achieving ≥VGPR after first-line therapy, stratified by CPCs proportion. [file Table5.docx]

**Supplementary Table 5. Rate of achieving ≥VGPR after first-line therapy, stratified by CPCs proportion.**

| **Subgroup** | **5-19% CPCs (N=30)** | **20% CPCs (N=16)** | ***P***-value |
| --- | --- | --- | --- |
| ≥VGPR achievement | 8 (26.7) | 6 (37.5) | 0.512 |
